# Treatment outcomes of pulpotomy versus pulpectomy in vital primary molars diagnosed with symptomatic irreversible pulpitis: protocol for a non-inferiority randomised controlled trial

**DOI:** 10.1186/s12903-024-04411-6

**Published:** 2024-05-28

**Authors:** Nebu Philip, Joe Mathew Cherian, Mebin George Mathew, Abi M. Thomas, Sunaina Jodhka, Nino John, Bharat Suneja, Mandeep Duggal

**Affiliations:** 1 College of Dental Medicine, QU Health, Qatar University, Doha, Qatar; 2 Department of Pedodontics and Preventive Dentistry, Christian Dental College, Ludhiana, India; 3 The Dentist Multi-Speciality Clinic, Ludhiana, India; 4 Primary Health Care Corporation, Doha, Qatar; 5 The Dental Care Centre, Ludhiana, India

**Keywords:** Pulpectomy, Pulpotomy, Vital primary molars, Irreversible pulpitis

## Abstract

**Background:**

Pulpectomy continues to be the standard treatment recommendation for management of vital primary molars diagnosed with symptomatic irreversible pulpitis. The recent decade has seen a paradigm shift in the treatment concepts of how vital mature permanent molars diagnosed with irreversible pulpitis can be more conservatively managed using vital pulp therapy techniques like pulpotomy. However, despite emerging evidence indicating similarities between primary and permanent tooth pulp response to dental caries, there is limited research on whether pulpotomy can be similarly used as a definitive treatment modality for vital primary teeth with irreversible pulpitis. This randomised controlled trial (RCT) aims to compare the treatment effectiveness of pulpotomy versus pulpectomy in management of vital primary molars diagnosed with symptomatic irreversible pulpitis over a two-year period.

**Methods/design:**

This clinical study is a parallel, two-armed, open label, non-inferiority RCT with a 1:1 allocation ratio between the experimental intervention arm (pulpotomy) and the active comparator arm (pulpectomy). Healthy cooperative children, between 4–9 years of age, who have painful primary molars with clinical symptoms typical of irreversible pulpitis will be recruited after obtaining informed consent from their parents/legal guardians. 50 vital primary molars clinically diagnosed with symptomatic irreversible pulpitis will be randomly distributed between the two treatment arms. The primary outcomes that will be assessed are clinical and radiographic success after six-months, one-year and two-years of the trial interventions. The influence of baseline pre-operative variables (age; gender; tooth type; site of caries; pre-operative furcal radiolucency; pre-operative pain intensity) and intra-operative factors (time taken to achieve haemostasis) on treatment outcomes will also be assessed. The secondary outcome evaluated will be the immediate (24 h and 7 d) post-operative pain relief afforded by the two treatment interventions.

**Discussion:**

This trial seeks to provide evidence on whether pulpotomy treatment can be no worse than the standard pulpectomy treatment for the management of symptomatic irreversible pulpitis in vital primary molars.

**Trial registration:**

ClinicalTrials.gov (NCT06183203). Registered on 30 January 2024.

## Background/rationale

Current guidelines from the American Academy of Pediatric Dentistry (AAPD) and the British Society of Paediatric Dentistry (BSPD) recommend pulpectomy as the standard treatment for vital primary molars diagnosed with irreversible pulpitis [1, 2]. Pulpectomy is a non-vital treatment procedure where the entire pulp tissue is extirpated, and the root canals debrided and shaped to receive a resorbable material to fill the canal space in the affected primary tooth. The main objectives of pulpectomy in painful primary molars are to keep them symptomless and functional, while also preserving the arch space until their exfoliation and replacement by their permanent successors [3]. Treatment indications for the more conservative pulpotomy procedure (where only the coronal pulp tissue is removed), are restricted to vital primary teeth diagnosed with reversible pulpitis or in case of carious/mechanical pulp exposures in symptomless vital primary teeth [1].

Traditionally, the clinical diagnosis of irreversible pulpitis in a deeply carious primary molar was based on patient reported symptoms of spontaneous lingering pain or pain that persists even after the removal of any provoking stimuli. Radiographic examination of such a tooth would reveal carious exposure of the pulp with or without furcal rarefaction. As conventional pulp sensibility tests are not very reliable in primary teeth, the vitality of a primary tooth diagnosed with irreversible pulpitis is usually ascertained intra-operatively after deroofing the pulp chamber, based on the colour and volume of the observed pulp tissue [4]. However, even if the pulp of such a tooth is judged to be vital, radicular pulp is still extirpated based on the clinical diagnosis of irreversible pulpitis. This treatment approach is adopted due to the traditional notion of the poor healing capacity of primary dental pulp [5]. Early histological studies seemed to indicate that primary teeth with a history of spontaneous pain are often associated with extensive degenerative changes extending into the radicular pulp and are poor candidates for vital pulpotomy techniques [6–8]. Another long-established rationale for performing pulpectomy in symptomatic vital primary teeth was the suggestion that primary tooth pulps exhibit a more pronounced and widespread inflammatory reaction to dental caries compared to permanent tooth pulps and hence would be poor candidates for pulpotomy [9].

The 21st century has seen an improved understanding of pulp biology, pulpal inflammatory processes, and the potential for pulp healing and repair. Histopathologic and histobacteriologic studies in cariously exposed “irreversibly” inflamed permanent tooth pulps have found healthy pulpal architecture, free from inflammation and bacteria, few millimetres away from the bacterially colonised necrotic tissue in the pulp chamber [10–12]. If the infected coronal pulp is completely removed, a favourable environment can be created for radicular pulpal healing as the immunoinflammatory cells get eliminated by apoptosis and the odontoblast-like cells induce dentine bridge formation. This contemporary understanding of pulp pathophysiology, along with the introduction of bioactive calcium silicate cements, has revolutionised treatment modalities for the management of irreversible pulpitis in mature permanent teeth, even when they are associated with apical periodontitis [13]. There is now abundant evidence from randomised controlled trials (RCTs) and systematic reviews favouring conservative vital pulp therapy in permanent teeth diagnosed with irreversible pulpitis [14–23].

A corresponding paradigm shift in the treatment approaches to “irreversibly” inflamed vital primary pulps has not been seen despite new evidence emerging regarding primary pulp biology. Immunocytochemical and vascular studies have now established that primary and permanent pulps have similar vascularity and showed a comparable degree of vasodilation and angiogenesis in response to the caries insult [24, 25]. The pulpal tissues in both carious primary and permanent teeth also showed similar neural changes when mounting a defense to deep caries [26]. Furthermore, although primary pulp tissue contains more immune cells in both normal and carious states, these immune cells appear to localize in a manner identical to that seen in permanent teeth pulp tissue [25]. These histological findings suggest the need to re-evaluate treatment approaches to primary dentition pulp therapy similar to what is currently underway within adult endodontics [5]. This is especially important as literature reports also suggest that clinical symptoms, radiographic findings, and conventional pulp sensibility tests do not provide accurate information about pulp status in primary teeth [27, 28].

There are several advantages if pulpotomy can be offered as a substitute to the standard pulpectomy treatment for the management of irreversible pulpitis in vital primary molars. The ribbon-like torturous root canal anatomy in primary molars makes pulpectomy a difficult procedure for general dentists to perform, often requiring a child in pain to be referred to a specialist paediatric dentist for definitive treatment. In many situations, specialist care may not be easily accessible, or parents may not be able to afford its costs. Consequently, some parents may opt to extract the painful primary molar tooth of their child, resulting in complications such as the loss of function and arch integrity. The technically simpler pulpotomy procedure is not only less challenging for general dentists but is also potentially less time-consuming and easier for young patients to tolerate, both very important advantages in the dental treatment of children. Moreover, the pulpotomized primary tooth retains the regenerative/repair potential of the remnant radicular pulp and the proprioceptive sensation of the tooth. Finally, treatment complications sometimes associated with pulpectomy (e.g., extrusion of root filling material beyond the primary root apex) can be avoided if pulpotomy is the treatment of choice.

There is limited research on whether pulpotomy can be offered as an alternate treatment to pulpectomy in vital primary molars diagnosed with irreversible pulpitis. A prospective cohort study of pulpotomy in 50 vital primary teeth with irreversible pulpitis reported 93% clinical success and 90% radiographic success after one year of treatment [29]. A more recent retrospective study found higher clinical success rates for pulpotomy (99%) than pulpectomy (88%) in primary molars with carious pulp exposures or symptomatic irreversible pulpitis over an 18-month period [30]. To the best of our knowledge, there have been no RCTs comparing treatment outcomes of pulpotomy vs. pulpectomy for management of irreversible pulpitis in vital primary molars. This study aims to fill this research gap and could be of great importance in initiating a paradigm shift in the treatment of vital primary molars diagnosed with irreversible pulpitis. The objectives and hypothesis of the study are detailed below.

### Objectives

- Compare the treatment effectiveness of full pulpotomy vs. single-visit pulpectomy in the management of vital primary molars diagnosed with symptomatic irreversible pulpitis based on the clinical and radiographic outcomes of the treated teeth over a two-year period.
- Assess influence of baseline pre-operative variables (age; gender; tooth type; site of caries; pre-operative furcal radiolucency; pre-operative pain intensity) and intra-operative factors (time taken to achieve haemostasis) on treatment outcomes.

### Hypothesis

The null hypothesis of the study is that the treatment outcomes of the experimental pulpotomy intervention will not be inferior to the highly successful outcomes of the standard pulpectomy treatment for vital primary molars diagnosed with irreversible pulpitis.

## Methods

### Trial design

This clinical study is a parallel, two-armed, open label, non-inferiority randomised controlled trial with a 1:1 allocation ratio between the experimental intervention arm (pulpotomy) and the active comparator arm (pulpectomy).

### Trial setting

This clinical trial will be conducted in the Paediatric Dentistry Department of Christian Dental College Ludhiana, India.

### Trial registration and protocol

As mandated by the International Committee of Medical Journal Editors (ICMJE) and jurisdictional legislation for conducting clinical trials in India, this trial has been prospectively registered on ClinicalTrials.gov and the Central Trial Registry—India (CTRI). Ethics approval has been obtained from the Institutional Ethics Committee of Christian Medical College Ludhiana (IEC Approval Number: 23-12-517/CDC) This clinical trial protocol follows the SPIRIT guidelines (Standard Protocol Items: Recommendations for Interventional Trials). Any protocol changes required in the future will be carried out on the ClinicalTrials.gov.

### Study participants: eligibility criteria

Healthy (ASA I and II) co-operative children (Frankl Scale + and + +) between the ages of 4 to 9 years will be invited to participate in the study if they have vital primary molars that meet the following *inclusion criteria*: (i) symptoms typical of irreversible pulpitis i.e., spontaneous unprovoked pain lasting few seconds to several hours in the days before the dental visit or pain that is exacerbated by hot and/or cold stimuli with the pain lingering even after removal of stimuli; (ii) post-deroofing the pulp chamber, vitality of the primary molar tooth is confirmed by visual inspection of pulpal haemorrhage (uniformly reddish pink vascular tissue indicates healthy pulp); (iii) post-coronal pulp amputation, radicular pulp health is confirmed by attainment of radicular pulp haemostasis within 8-min of compression with a 5% sodium hypochlorite (NaOCl)-dampened cotton pellet; (iv) the primary molar is restorable with stainless steel crown; and iv) any physiologic root resorption, if present, is less than 1/3 the normal root length.

The *exclusion criteria* include: (i) clinical examination reveals signs of pulpal infection i.e., pathologic tooth mobility, parulis/fistula, or soft tissue swelling; (ii) pre-operative periapical radiograph suggests presence of furcal radiolucency more than ½ the furcation to periapical area; (iii) pre-operative periapical radiograph suggests presence of periapical radiolucency; (iv) pre-operative periapical radiograph suggests presence of pathological root resorption (v) post-deroofing the pulp chamber, visual examination of pulp tissue reveals signs of necrosis i.e., avascular/minimally bleeding pulp tissue or yellowish necrotic areas/purulent exudate; (vi) post-coronal pulp amputation, there are signs of extensive radicular pulp inflammation i.e., bleeding continues even after 8-min compression with NaOCl-soaked cotton pellet; and (vii) parents not willing to place full coverage crowns post-treatment; and (vii) if both primary molars in the quadrant are painful and clinical diagnosis of irreversible pulpitis between the teeth is not sharply defined.

### Trial interventions

The proposed trial interventions, pulpotomy and pulpectomy, will adhere to standard clinical procedural guidelines. Treatment group allocation of the consenting trial participants to one of the two intervention groups will be carried out only after intra-operative confirmation of pulp vitality and achievement of radicular pulp haemostasis. The choice of the treatment group (pulpotomy or pulpectomy) will be revealed to the operating clinician by an independent allocator only after coronal pulp amputation and confirmation of pulp vitality and haemostasis. Participants with pulp assessed to be non-vital or with uncontrolled bleeding from the pulp stumps will be excluded prior to allocation.

**Fig. 1:**
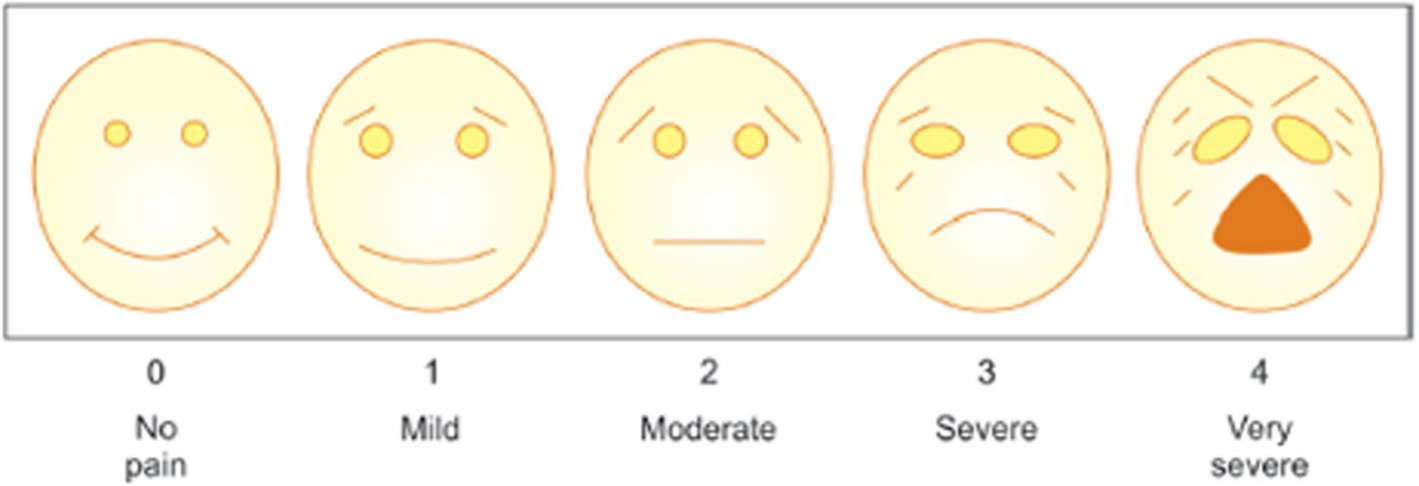
Visual analogue scale for pain assessment in children

Standard clinical protocol for the pulpotomy group:

1. Detailed history of symptoms will be verified and a thorough clinical examination shall be performed.
2. Pre-operative pain intensity will be recorded using the validated five-face visual analogue scale (VAS) commonly used for pain intensity measurement in children (Fig. 1). After giving explanations of the VAS, children will be requested to select one of the faces that best reflect their pain intensity.
3. A standard pre-operative periapical radiograph will be taken using a paralleling technique and a film holder device. Care will be taken to ensure that the radiographic image extends beyond the root tip of the primary molar tooth and there is no evidence of distortion, overlap, or processing errors.
4. Local anaesthesia will be achieved with 2% lidocaine or 4% articaine containing 1:100,000 epinephrine and the tooth will be isolated under rubber dam.
5. To minimize further bacterial contamination of the pulp, carious tissues will be progressively removed, starting at the periphery of the cavity and then over the pulp chamber roof.
6. Once pulp chamber deroofing is complete, a fresh sterile bur will be used to remove all coronal pulp tissue to the level of the root canal orifices, under copious water irrigation. During this step, intra-operative assessment of pulp vitality will also be carried out. Healthy vital pulp will present as uniformly reddish pink vascular tissue, while non-vital necrotic pulp will present as dark avascular tissue with minimal bleeding or as yellowish liquefied areas. If pulp is judged to be necrotic, the tooth will be excluded from the study. Further treatment of such excluded teeth will be delivered following local management protocols.
7. Haemostasis and disinfection of the radicular pulp tissue will be carried out by compressing a 5% NaOCl-soaked sterile cotton pellet over the pulp stumps for up to 8-min. Up to three attempts will be made to control pulp stump bleeding using moderate pressure applied with the NaOCl-dampened cotton pellet. Pulpal haemostasis will be assessed at three timepoints (3-, 6-, and 8-min) and the pulpotomy medicament placed over the pulp stumps as soon as haemostasis is achieved. If bleeding persists beyond 8-min, the radicular pulp shall be considered irreversibly inflamed, and the tooth will be excluded from the study. Further treatment of such excluded teeth will be delivered following local management protocols.
8. Once haemostasis is achieved, 2 mm of a pre-mixed mineral trioxide aggregate (MTA) (NeoPutty, NuSmile, Houston, TX, U.S.A) medicament will be directly adapted over the pulp stumps ensuring that there is no porosity or any excess cement on the pulp chamber walls.
9. The access cavity will be restored with a bulk-fill high-strength glass ionomer cement (3M ESPE Ketac Molar, Seefeld, Germany). Post-operative radiograph will be taken to confirm fidelity to the pulpotomy procedure.
10. The pulpotomy-treated tooth will be prepared for receiving a full coverage stainless steel crown (SSC) one week after the pulpotomy procedure.

Standard clinical protocol for the pulpectomy group:

The initial seven procedural steps of the pulpotomy treatment group shall be followed in the pulpectomy treatment group too. Once vitality is confirmed and haemostasis achieved, the entire radicular pulp will be extirpated, and the root canals prepared as described below:

1. Radicular pulp will be extirpated from all the root canals using appropriate endodontic instruments.
2. Maintaining a working length 1–1.5 mm short of the radiographic apex, chemo-mechanical preparation of the canals will be done using a series of 21 mm long Kerr-type endodontic files up to file no. 30 or 35 (depending on the initial size of the canal). Regular irrigation of the prepared canals will be carried out using 2.5% NaOCl alternating with saline.
3. Sterile absorbent paper points will be used to dry the root canals prior to obturation with root filling material that contains zinc oxide-eugenol, Ca(OH)_2_, and iodoform (Endoflas; Sanlor Laboratories, Cali, Colombia). The Endoflas material that is available in a powder-liquid form will be mixed into a medium consistency such that it forms a 1 cm string when lifted from the glass slab with a cement spatula. A reamer coated with the mixed Endoflas will be used to insert the material into the prepared root canals and pressure applied with a sterile cotton pellet to ensure optimal canal obturation.
4. Access cavity will be restored with a bulk-fill high-strength glass ionomer cement (3M ESPE Ketac Molar, Seefeld, Germany). Post-operative radiograph will be taken to confirm fidelity to the pulpectomy procedure.
5. The pulpectomy-treated tooth will be prepared for receiving a full coverage SSC one week after the pulpectomy.

Post-operative instructions for both pulpotomy and pulpectomy treatment groups will advise patients to take analgesics if necessary and to return if the pain is intolerable.

### Follow-up timeline

Children enrolled in the study will be followed from the trial intervention until the end of the study (two years post-treatment). Trial participants and their parents/guardians will be initially contacted by phone 24 h after the treatment intervention to record their post-operative pain intensity score using the VAS. Post-operative pain intensity score will also be recorded 7 d after the treatment intervention before the placement of the SSC. Clinical and radiographic outcome data of the treated tooth will be recorded at 6 months, 12 months, and 24 months post-treatment. Children participating in the trial will be advised to continue their routine dental visits, with the recall period determined by their caries risk status. Any symptomatic visits to the dentist outside these follow-up timepoints will be identified and reason for the same recorded on the trial record form.

### Trial outcomes

#### Primary outcomes

Clinical outcomes will be assessed by a single experienced paediatric dentist who shall be blinded to the treatment allocation. Radiographic outcomes will be assessed independently by two experienced paediatric dentists under optimum viewing conditions.

*Clinical success* of the treated tooth will be determined if all the following conditions are met:

- Absence of pain or discomfort
- Absence of tenderness on percussion and palpation
- Absence of any associated parulis/fistula
- Absence of any associated soft tissue swelling
- Absence of any associated pathological mobility

*Radiographic success* will be determined as follows:

- Absence of pathosis on recall periapical radiograph i.e., no signs of pathological internal/external root resorption or new furcal/periapical lesions.
- Complete radiographic healing or reduction/no change in size of the any pre-operative furcal rarefaction. Teeth that show an increase in the size of any pre-operative furcal radiolucency will be considered as failed.

#### Secondary outcomes

- *Immediate post-treatment pain relief:* post-operative pain felt by the child in the immediate aftermath of the trial interventions will recorded at two time-points (24 h and 7 d) using the VAS. The pain scores recorded will be used to evaluate pain reduction afforded by the two trial interventions.

### Target sample size

Sample size determination for this binary outcome non-inferiority randomised trial was calculated using a computer-generated system available online (https://www.sealedenvelope.com/power/binary-noninferior/). Based on previous clinical studies [29–31], a 95% success rate was assumed for both treatment interventions along with a 17% non-inferiority margin. Even an overall success rate of 78% for the experimental pulpotomy intervention would be considered non-inferior to the standard pulpectomy intervention. To obtain a power of 80% (β = 0.20) and a 2-sided α equal to 0.05, approximately 42 patients for both groups will be needed. To compensate for loss during follow-up and other causes of attrition, the targeted sample size will be 50 vital primary molar teeth diagnosed with irreversible pulpitis, distributed equally between the two trial treatment arms.

### Recruitment

**Fig. 2:**
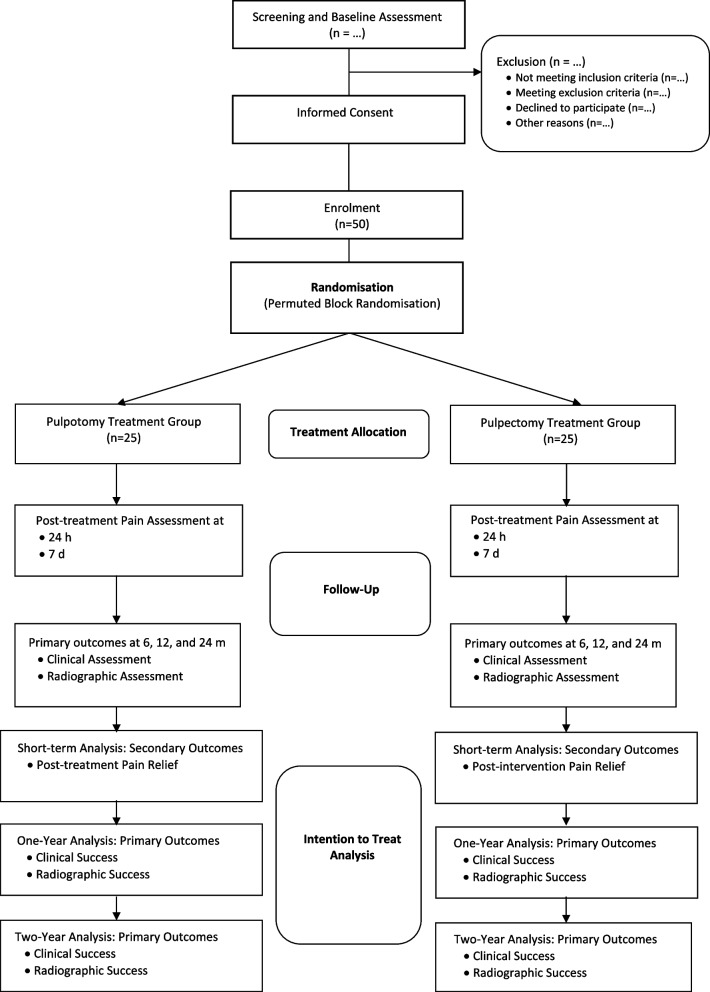
Trial schematic of the proposed study

All children in the 4 to 9-year age group who report to the Paediatric Dentistry Departments of Christian Dental College with symptoms typical of irreversible pulpitis in their primary molars will be considered for recruitment. Participant recruitment will be based on the defined inclusion and exclusion criteria. Recruitment is planned to start in February 2024 and expected to last for up to 8–10 months. No financial or non-financial incentives will be provided to trial investigators or participants for enrolment. A trial schematic outlining the screening, recruitment, randomisation, allocation, follow-up time-points, and planned data analysis is shown in Fig. 2.

### Randomisation and sequence generation

The randomization list will be generated before participant enrolment. Permuted block randomisation will be used to randomly assign one of the two treatment interventions to consenting trial participants who meet all the inclusion/exclusion criteria. Block lengths will vary according to a sequence generated by an external researcher. The on-site trial investigators will be blinded to the block sizes and the randomization sequence.

### Allocation concealment

The generated randomization sequence will be distributed in sequentially numbered opaque sealed envelopes (SNOSE). An independent allocator, not involved in the assessment and recruitment of the patient, will open the sequentially numbered envelope, and inform the clinical investigator which treatment group the patient is assigned to. Patient details will be written on the outside of the envelopes (that contain carbon paper overlying the treatment allocation paper), so that patient names are transferred to the allocation paper before the envelope is opened and can be audited at the end of the trial.

### Blinding

Blinding of trial participants or clinical investigators will not be possible due to evident differences between the two trial interventions. However, post-operative clinical outcomes will be assessed by an experienced paediatric dentist who is blinded to the treatment allocation, while the radiographic outcomes will be independently assessed by two different paediatric dentists. An external researcher, not directly involved in the clinical part of the study, will generate the randomization sequence, provide the SNOSE for treatment group allocation, and perform the statistical analysis of the de-identified data.

### Data collection, management, and confidentiality

Relevant demographic, clinical, radiographic, and pain intensity data will be collected at baseline and subsequently at the follow-up time points described earlier. All data collected will be recorded electronically at the trial site using unambiguous, standardized terminology and abbreviations to avoid misinterpretation. Checks will be applied at the time of data entry before data is committed to the electronic database to ensure accuracy of entered trial data. Any later modifications to data entered in the database will be documented. All participant data will be de-identified and stored on an institutional password protected computer. Data will only be accessible to authorized members of the research team. Data will be treated confidentially and stored for 3 years before being destroyed.

### Statistical methods

All analyses will use the ‘intention to treat’ principle. Baseline demographic and clinical data will be summarised for each treatment group. Binary logistic regression models will be applied to analyse all outcome measures stated in the protocol adjusting for covariates thought to be of prognostic importance. Appropriate parametric or non-parametric tests will be used to determine any significant relationship between treatment outcomes and variables in the study (age, gender, tooth/caries type, and pre-operative furcal rarefaction/pain intensity). For all statistical tests, we will use 2-sided *p*-values with α ≤ 0.05 and the treatment effect estimates will be presented with 95% confidence intervals. Up-to-date versions of SPSS (Chicago, IL, USA) will be used to conduct the statistical analysis.

### Expected risks

Whilst performing pulpotomy in vital primary molars diagnosed with irreversible pulpitis is a novel treatment, it is more conservative than the standard pulpectomy treatment. Pulpotomy can be considered as the first stage of pulpectomy and an effective emergency measure to manage pain associated with an infected pulp. Thus, we do not anticipate any significant safety concerns with the treatment interventions of this trial. The clinicians taking part in the trial are fully trained in the pulpotomy and pulpectomy techniques and all subjects enrolled in the trial will receive the usual standard of care treatment during and following the trial intervention. The trial site principal investigators (PIs) will record adverse events (AEs) and serious adverse events (SAEs) only if they have a reasonable casual relationship with the treatment interventions of the study. Rare but potential AEs and SAEs include perforation and hypochlorite injury. Patients who continue to experience pain following trial interventions will be able to access emergency care to address their symptoms. Trial site PIs will report AEs/SAEs to the trial co-ordinator who will escalate them as deemed necessary. AE reporting period for this trial begins upon enrollment and ends at the two-year follow-up period for participants.

### Ethical considerations

Participants and their guardians will be invited to enrol in the trial voluntarily and will be assured that should they chose not to participate the standard treatment will be offered as normal with no prejudice. Study participants will be recruited to the trial only after obtaining the child’s verbal assent and their parents or guardians sign the Informed Consent Form containing detailed information about the study. Patients and their guardians will have the opportunity to ask questions and take any additional time they may need prior to their decision to participate. Participants and their guardians will be given the option of withdrawing from the study should they choose to do so at any time in the future.

### Dissemination

Study results will be reported according to the Consolidated Standards Of Reporting Trials (CONSORT) guidelines through conference presentations and scientific articles in peer-reviewed journals. Authorship of the publications emerging from this study will be determined based on the ICMJE guidelines. Any significant amendments to this study protocol will be reported when the study results are disseminated.

## Discussion

The proposed study is unique in that for the first time a non-inferiority RCT is evaluating whether the experimental pulpotomy treatment can prove to be no worse than the standard pulpectomy treatment for the management of symptomatic irreversible pulpitis in vital primary molars. Considering that pulpotomy has been shown to have highly successful treatment outcomes in vital mature permanent molars diagnosed with irreversible pulpitis, there exists the possibility of similar successful outcomes in primary molars too. Consequently, this trial has the potential to produce new knowledge and add information to an area that is currently under researched.

It is important to highlight that the root filling material proposed to be used in the pulpectomy intervention group (Endoflas) has among the highest success rates reported for primary root canal filling materials [31–35]. Similarly, the new generation MTA product proposed to be used in the pulpotomy intervention group not only retains the biocompatibility, immunomodulatory, osteogenic, and sealing properties of traditional MTA, but being a resin-free pre-mixed bioceramic has superior handling characteristics, faster washout and setting times [36, 37]. The NaOCl proposed to be used as the haemostatic/disinfection agent in the pulpotomy intervention group demonstrated comparable clinical/radiographic success to traditional pulpotomy medicaments like formocresol and ferric sulphate without their drawbacks [38–40]. The two-year follow-up period proposed in this study should be adequate to assess treatment success of the trial interventions in primary molar teeth. Studies with longer follow-up periods can be planned based on the initial results of this study.

If the hypothesis of the proposed study is confirmed, it will support the clinical application of more conservative treatment approaches to vital primary teeth with irreversible pulpitis. Such an approach has the potential to cause less pain and discomfort to paediatric patients, have a shorter chair-time, and lower the technical difficulty and cost of managing complicated pulp conditions in vital primary molars.

## Data Availability

Deidentified data sets that will be generated and/or analysed in the current study shall be made available from the corresponding author upon reasonable request. A summary of final findings, in layman’s language, will be made available to the trial participants.

## Abbreviations

AAPD: American Academy of Pediatric Dentistry
AE: Adverse Event
ASA: American Society of Anesthesiology
BSPD: British Society of Paediatric Dentistry
CONSORT: Consolidated Standards Of Reporting Trials
CTRI: Central Trial Registry India
GIC: Glass Ionomer Cement
ICMJE: International Committee of Medical Journal Editors
MTA: Mineral Trioxide Aggregate
RCT: Randomised Controlled Trials
SAE: Serious Adverse Event
SNOSE: Sequentially Numbered Opaque Sealed Envelopes
SPIRIT: Standard Protocol Items: Recommendations for Interventional Trials
VAS: Visual Analogue Scale
